# Hypertonic saline for fluid resuscitation after cardiac surgery (HERACLES): study protocol for a preliminary randomised controlled clinical trial

**DOI:** 10.1186/s13063-019-3420-6

**Published:** 2019-06-14

**Authors:** Carmen A. Pfortmueller, Anna S. Messmer, Benjamin Hess, David Reineke, Laura Jakob, Stefanie Wenger, Jan Waskowski, Patrick Zuercher, Frederik Stoehr, Gabor Erdoes, Markus M. Luedi, Stephan M. Jakob, Lars Englberger, Joerg C. Schefold

**Affiliations:** 1 Department of Intensive Care Medicine, Inselspital, Bern University Hospital, University of Bern, Freiburgstrasse 18, CH-3010 Bern, Switzerland; 2 Department of Anaesthesiology and Pain Medicine, Inselspital, Bern University Hospital, University of Bern, Bern, Switzerland; 3 Department of Cardiovascular Surgery, Inselspital, Bern University Hospital, University of Bern, Bern, Switzerland

**Keywords:** Hypertonic saline, Normal saline, Fluid resuscitation, Cardiac surgery, Cardiosurgery, Ischaemic heart disease, Valvular heart disease, Fluid balance, Critical illness, ICU

## Abstract

**Background:**

Intraoperative and postoperative management of cardiac surgery patients is complex, involving the application of differential vasopressors and volume therapy. It has been shown that a positive fluid balance has a major impact on postoperative outcome. Today, the advantages and disadvantages of buffered crystalloid solutes are a topic of controversy, with no consensus being reached so far. The use of hypertonic saline (HS) has shown promising results with respect to lower total fluid balance and postoperative weight gain in critically ill patients in preliminary studies. However, collection of more data on HS in critically ill patients seems warranted. This preliminary study aims to investigate whether fluid resuscitation using HS in patients following cardiac surgery results in less total fluid volume being administered.

**Methods:**

In a prospective double-blind randomised controlled clinical trial, we aim to recruit 96 patients undergoing elective cardiac surgery for ischaemic and/or valvular heart disease. After postoperative admission to the intensive care unit (ICU), patients will be randomly assigned to receive 5 ml/kg ideal body weight HS (7.3% NaCl) or normal saline (NS, 0.9% NaCl) infused within 60 min. Blood and urine samples will be collected preoperatively and postoperatively up to day 6 to assess changes in renal, cardiac, inflammatory, acid-base, and electrolyte parameters. Additionally, we will perform renal ultrasonography studies to assess renal blood flow before, during, and after infusion, and we will measure total body water using preoperative and postoperative body composition analysis (bioimpedance). Patients will be followed up for 90 days.

**Discussion:**

The key objective of this study is to assess the cumulative amount of fluid administered in the intervention (HS) group versus control (NS) group during the ICU stay. In this preliminary, prospective, randomised controlled clinical trial we will test the hypothesis that use of HS results in less total fluids infused and less postoperative weight gain when compared to the standard of intensive care in cardiac surgery patients.

**Trial registration:**

ClinicalTrials.gov, NCT03280745. Registered on 12 September 2017.

**Electronic supplementary material:**

The online version of this article (10.1186/s13063-019-3420-6) contains supplementary material, which is available to authorized users.

## Background

Cardiovascular surgery for ischaemic or valvular heart disease has become a major approach in daily practice in industrialised countries, with more than 100,000 surgeries performed in Germany per year [1]. Perioperative management of cardiac surgery patients remains complex; challenges for anaesthesiologists and intensive care physicians arise from specific characteristics of the underlying cardiac disease, the complexity of the surgical intervention, and the pathophysiological impact of extracorporeal circulation [2]. Furthermore, cardiac surgery alters fluid balance significantly, generates a systemic inflammatory response that increases oxygen consumption, and is associated with changes in cardiac output and oxygen delivery [3, 4].

Recommendations for fluid strategies have changed considerably in recent years [1]. Despite the ongoing debate over the role of colloids in patients undergoing cardiac surgery [4–6], growing doubt with regard to the safety and efficiency of colloid solutes has led to increased use of crystalloid solutes [1, 7, 8]. According to a recent large multicentre survey conducted in Germany, crystalloids are considered the fluid of choice for intraoperative fluid therapy, cardiopulmonary bypass circuit priming, and post-surgery intensive care [1].

Critically ill patients, and especially patients undergoing cardiac surgery, receive considerable amounts of intravenously administered fluid [9, 10], which often results in fluid overload. Despite common perception, fluid overload is not a benign occurrence, with mounting data demonstrating an association with prolonged intensive care unit (ICU) and hospital length of stay and increased mortality [11, 12]. In addition, fluid overload (defined as a ≥ 10% increase in body weight) [13] has been associated with increased perioperative complications [14, 15] and decreased gastrointestinal function [9, 16].

Fluid resuscitation using a bolus or continuous infusion of hypertonic saline (HS) has been used for more than 30 years [9, 17, 18]. HS expands intravascular volume by shifting fluid from the extravascular space [19, 20], thus increasing preload [21, 22]. Compared to conventional artificial plasma expanders or human plasma, HS is considered inexpensive, rapidly available, and without risk of anaphylaxis or transmission of infectious disease [22]. Despite potential physiological advantages, HS has been used less in recent years, which may at least partially be due to previous use of mixed solutions (e.g. HS with colloids). Currently, studies assessing the use of HS for fluid resuscitation in critical illness are scarce and heterogeneous regarding effects on clinical outcomes. However, several studies have shown that infusion of HS results in less volume administered [22–26], favourable total fluid balance [22–24, 26], and less weight gain after surgical procedures when compared to normally used crystalloids [23, 27]. Thus, fluid resuscitation with HS infusion may provide benefits in selected critically ill patients. In patients undergoing cardiac surgery, only one study was conducted with the primary outcome of fluid status after surgery [27]. It showed promising results in terms of less total volume infused, less postoperative weight gain, and increased postoperative diuresis. However, these findings await confirmation in a larger population.

We propose a preliminary prospective, single-centre, randomised controlled clinical trial on the use of HS (7.3% NaCl) versus NS (0.9% NaCl) in addition to conventional fluid resuscitation with lactated Ringer’s solution in patients after cardiac surgery. We will test the hypothesis that use of HS results in less total volume infused and less postoperative weight gain when compared to fluid resuscitation with the standard of care in patients after cardiac surgery.

## Objectives

### Primary objective

This preliminary study aims to investigate whether fluid resuscitation using HS in patients following cardiac surgery results in less total fluid volume administered.

### Secondary objectives

Secondary objectives are to investigate whether the use of HS results in less postoperative weight gain, reduced cumulative vasopressor dose and shorter time on vasopressors after cardiac surgery, as well as increased urinary output.

### Exploratory outcomes

Further, we will assess whether use of HS results in a change in acid-base parameters and electrolyte levels as well as inflammatory/immune, cardiac, and renal function indices. Differences in clinical outcome comparators, including body composition analysis (water balance) using bioimpedance, need for renal replacement therapy, time on the ventilator, length of ICU and hospital stay, as well as ICU and in-hospital mortality and 30-day and 90-day mortality, will also be analysed.

## Methods

The “Hypertonic saline for fluid resuscitation after cardiac surgery (HERACLES)” study is a single-centre prospective double-blind randomised controlled clinical trial taking place in a tertiary care university hospital (Department of Intensive Care Medicine, Inselspital, Bern University Hospital, University of Bern, Switzerland). Recruitment started February 27, 2018 (ongoing); the current protocol version is V1.3, dated March 1, 2018.

All patients undergoing cardiac surgery at our institution will be screened according to the predefined inclusion and exclusion criteria before written informed consent is obtained. Prior to enrolment, the investigators will explain to each participant the nature of the study, its purpose, the procedures involved, the expected duration, and the potential risks and benefits. To give consent to participate, the patient and the investigator or his/her designee will sign an informed consent form (ICF). Thereafter, the patient will be enrolled in the study. Study procedures are performed according to the planned schedule with adherence to visit intervals.

**Fig. 1:**
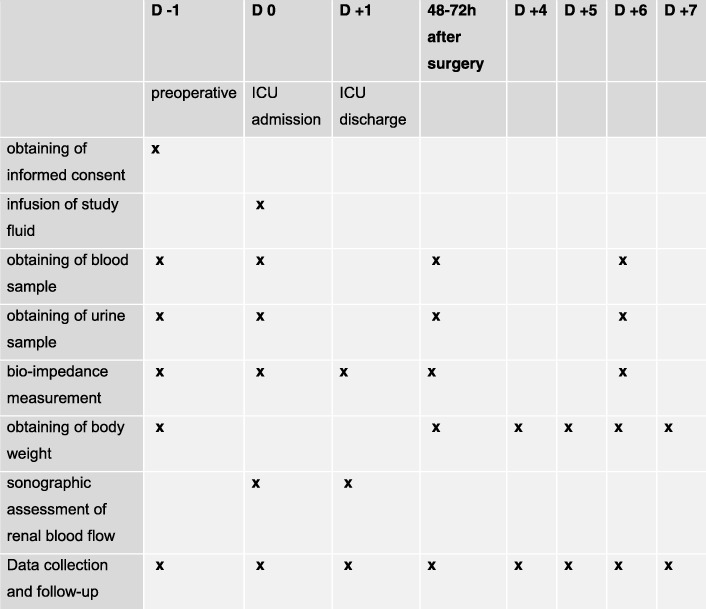
SPIRIT figure indicating visits and study-specific assessments

A Standard Protocol Items: Recommendations for Interventional Trials (SPIRIT) checklist is provided in Additional file 1. A schedule based on SPIRIT recommendations is shown in Fig. 1.

### Approvals

This study has been approved by the cantonal committee responsible for ethical research in humans (Kantonale Ethikkommission, KEK, Bern, Switzerland, approval number 2017-00647). Written informed consent will be obtained from all participants.

### Population

We aim to include a total of 96 patients (n = 48 per group) undergoing elective cardiac surgery for ischaemic and/or valvular heart disease and receiving the allocated study investigational medicinal product (IMP).

#### Inclusion criteria

Patients aged ≥ 18 years who provide informed consent meet the inclusion criteria.

#### Exclusion criteria

The exclusion criteria are as follows:

- Inability to provide informed consent
- Pregnancy or breastfeeding
- Preoperative left ventricular ejection fraction (LVEF) < 30%
- Pre-existing renal insufficiency with an estimated glomerular filtration rate (eGFR) < 30 ml/min/1.73 m^2^
- Postoperative mechanical circulatory support devices such as left ventricular assist device (LVAD), intra-aortic balloon pump (IABP), Impella, or extracorporeal membrane oxygenation (ECMO) device
- Pre-existing serum sodium level of > 145 mmol/L or < 135 mmol/L
- Pre-existing serum chloraemia > 111 mmol/L or < 96 mmol/L
- Systemic steroid therapy (at any dose at time of inclusion)
- Chronic liver disease (bilirubin > 3 mg/dl)
- Any signs of infection or sepsis defined as clear clinical evidence for active infection or current antibiotic therapy

### Randomisation and blinding

All patients undergoing cardiac surgery and meeting the eligibility criteria as defined above will be enrolled in the study and randomised to either the HS group (7.3% NaCl) or the NS group (0.9% NaCl). Computerised randomisation using a variable block size [2, 4, 6] will be performed by a member of the study team not involved in any part of treatment (neither nurse, resident, nor supervisor). A box with the study vials and study infusion will be given to the treating intensive care physician with the patient’s number in the study. Participants, care providers, investigators, and outcome assessors will be blinded to study group assignment. A list for emergency un-blinding of the study will be held by a designated study quality manager.

### Management of study material

Boxes with the study numbers containing unlabelled infusions will be prepared for all patients prior to study start. One box will contain a 1 × 500 ml + 1 × 100 ml flask of 0.9% NaCl (B. Braun, Sempach, Switzerland) and 20 unlabelled vials of either 0.9% or 23.4% saline (Bichsel, Interlaken, Switzerland). For patients who weigh < 111 kg, 100 ml 0.9% NaCl of the 500-ml 0.9% NaCl flask will be removed by syringe and 16 vials will be added to the remaining 400 ml 0.9% NaCl, to create either a study infusion of 0.9% saline or 7.3% saline.

For patients who weigh > 110 kg, we will prepare a 500-ml 0.9% NaCl flask as described for patients ≤ 110 kg and 1 ×100 ml 0.9% NaCl to which 4 vials of either study infusion are added. All patients will receive 5 ml/kg of the study infusion over 60 min. The weight-adjusted amount of study fluid will be prepared bedside by the treating ICU nurse and given at 5 ml/kg body weight over 60 min via infusion pumps through a central venous line.

### Study conduct

Patients included in the trial will receive standard monitoring for cardiac surgical patients in the ICU. We routinely monitor oxygen saturation, heart rate, invasive arterial blood pressure, electrocardiography, temperature, urine output, and central venous pressure.

Echocardiography will be performed preoperatively and intraoperatively. If patients are considered haemodynamically unstable, they receive a pulmonary artery catheter (PAC) according to the hospital’s standard operating procedures (SOPs). Blood gas analysis parameters and mixed venous oxygen saturation will be measured according to clinical routine or as indicated at the discretion of the attending ICU physician.

#### General management

Maintenance of anaesthesia as well as haemodynamic management during and after cardiopulmonary bypass are standardised and protocol-driven according to the hospital’s SOPs. All patients are sedated and ventilated during transfer to the ICU. A blood gas analysis is performed before the start of the study intervention and 1 h after study infusion termination. Blood gas analyses are performed every 2 h for the next 6 h and thereafter according to clinical routine. Additionally, renal ultrasonography is performed to assess renal blood flow prior to infusion as well as at 1 h and 2 h after termination of the study fluid and on the first postoperative day.

#### Fluid management and haemodynamic protocol in the ICU

On admission to the ICU, patients will receive 5 ml/kg ideal body weight of HS (7.3% NaCl) or NS (0.9% NaCl) by infusion pump for 60 min. Thereafter, ICU treatment will continue according to clinical routine.

If needed, norepinephrine is the first-choice vasopressor and will be titrated to a mean arterial pressure (MAP) judged adequate for the respective patient depending on age and history of hypertension and kidney injury. The range of target MAP is typically between 55 and 65 mmHg. Epinephrine will be added when an additional agent is needed to maintain adequate blood pressure, and if necessary norepinephrine will be exchanged for epinephrine. Dopamine is not used. In the presence of myocardial dysfunction — as suggested by increased cardiac filling pressures and low cardiac output — a dobutamine infusion will be administered. If patients become hypertensive, afterload will be reduced by nitroprusside natrium infusion.

#### Management of biological material

Two samples of blood as well as a urine sample will be drawn and stored at − 80 °C at the four defined time points (Fig. 1). Coded samples will be stored by the Center of Laboratory Medicine, Inselspital, Bern University Hospital, Switzerland.

### Safety measures during study conduct

The infusion of HS can lead to several side effects including electrolyte disturbances (hypernatraemia, hyperchloraemia, hypokalaemia), occurrence of acidosis, and development of oedema. Hence the following safety measures are taken: Before the start of the study intervention, in the ICU a secondary screening will be performed. First, a blood gas analysis will be done before the start of the study intervention to ensure a serum sodium concentration postoperatively below 145 mmol/L and a serum chloride concentration below 107 mmol/L. Second, a doctor of the research team will be contacted if intraoperative complications such as massive bleeding, severe haemodynamic turbulence, or intraoperative myocardial infraction occurred. Based on the blood gas analysis results and/or the medical history, it will be decided if the study is to proceed. Patients admitted from the ICU with cardiac assist devices such as an IABP or an ECMO device or electrolyte levels outside the normal range will be dropped from the study before receiving the study intervention.

If the study is started, a blood gas analysis will be performed 1 h after study infusion termination. For the first 20 patients we will perform a blood gas analysis after 30 min and 60 min of study infusion to check serum sodium levels; thereafter, if the sodium levels stay below 155 mmol/L during infusion of the study fluid, this additional blood gas analysis will be omitted. One hour after termination of the study infusion an additional blood gas analysis will be performed; thereafter, blood gas analyses will be performed every 2 h for the next 6 h and thereafter according to clinical routine.

If the serum sodium level is above 155 mmol/L or the serum chloride level is above 125 mmol/L at any time point, the infusion of the study medication will be stopped.

### Study parameters

We will obtain baseline data (age, gender, height, weight, BMI), baseline comorbidities, risk scores, and preoperative and postoperative routine laboratory parameters in all patients. Additionally we will perform sonographic assessment of renal blood flow and assess body composition (bioimpedance) at predefined endpoints.

All types of fluids and the cumulative amounts (blood products included), daily body weight, and cumulative doses of medication will be documented. Furthermore, the need for renal replacement therapy, length of mechanical ventilation, length of ICU and hospital stays, and mortality will be assessed.

### Outcome measures

#### Primary endpoint

The primary endpoint is the difference in total cumulative fluid volume administered during ICU stay between patients receiving HS and patients receiving NS following elective cardiac surgery.

#### Secondary endpoints

Secondary endpoints are to investigate whether use of HS results in:

- Reduced cumulative vasopressor dose and shorter time on vasopressors after cardiac surgery
  - Differences in urinary output and postoperative weight gains between the two groups.

#### Exploratory outcomes

Exploratory outcomes are to assess whether the use of HS results in:

- A change in acid-base parameters and electrolyte levels as well as inflammatory/immune function indices
- Differences in clinical outcome comparators, including body composition analysis (water balance) using bioimpedance, need for renal replacement therapy, time on the ventilator, length of ICU and hospital stays, as well as ICU mortality and in-hospital mortality. We will also analyse 30-day and 90-day mortality.

### Study timetable

The timing of assessment of study parameters is outlined in Fig. 1.

### End of study

For included patients, the study will start with admission to the ICU and will end after day 6. Thereafter, patients will be followed up for secondary endpoints. Patients will not be monitored further if they are readmitted to the ICU.

### Statistics

Statistical evaluation will be conducted in the intent-to-treat population (carry forward last value method).

Descriptive presentation of the recorded data will be based on a scale type. Nominal/ordinal scales include cell frequencies at each level, missing data, valid number, and total number. Interval/proportional scales include arithmetic mean, standard deviation, median, spread, minimum, maximum, and valid number. In addition to the summary of the data using statistical parameters, descriptions will be provided individually for the total population as well as for both groups.

#### Statistical methods

This is a classic two-group study with parallel groups. Whether HS results in less total fluid volume will be assessed by the Mann-Whitney *U* test. Interval variables between groups will be compared using Student’s *t* test or the Mann-Whitney *U* test as appropriate. Fisher’s test for exact probability will be used for the nominal scale data. In the event of an uneven baseline situation, multivariable regression (linear regression for interval outcomes and logistic regression for dichotomous outcomes) will be used for adjustment of baseline differences.

#### Estimation of sample size

Sample size calculation was based upon fluid data from the Hemacetat study (NCT02895659) [28], conducted at our institution with the same patient collective. Preliminary data showed that patients received a mean of 3381 ml fluid during their ICU stay, with a standard deviation of 1734 ml. A clinically relevant effect size was determined to be a reduction of 1000 ml fluid (30%).

#### Rationale for effect size

Although several studies reported reduced infusion volumes when HS was used for fluid resuscitation [29], there is a large heterogeneity in studies on HS use, with widely varying amounts of sodium administered. In consequence, it was not possible to determine the amount of fluid to be saved when HS is used based on the available literature. Fluid overload is harmful in patients undergoing cardiac surgery and related to adverse outcomes [30–32] and thus should be avoided. However, the ideal amount of fluid for these patients is not yet clarified. In a pragmatic approach, we hence determined that it would be clinically beneficial if the total amount of fluid administered intravenously in the ICU (average 3381 ml at our institution) could be lowered by 1 L or 30%.

Modelling based on values of this study was achieved with the Power and Sample Size Calculator provided by the Statistical Institute of the Medical University of Vienna (http://statistics.msi.meduniwien.ac.at) and cross-checked on http://powerandsamplesize.com. The following constellations were obtained with regard to the required case numbers: n = 48 with a power of 80% and an α error of 0.05. Based on these results, a number of *n* = 48 patients with an IMP for each group, for a total number of 96 patients with an IMP, was determined to be sufficient.

### Ethical considerations

The study will be carried out in accordance with the protocol and with principles enunciated in the current version of the Declaration of Helsinki and the International Conference on Harmonisation (ICH) guidelines for Good Clinical Practice (GCP). The KEK and regulatory authorities will receive annual safety and interim reports and be informed if the study is discontinued, in agreement with local requirements. The KEK will be informed within 90 days if the study is discontinued.

All patients must give consent to participate in the study after receiving a detailed explanation of the potential benefits and harms. Patient information material as well as the consent form are included in the appendix of the study protocol. The KEK will receive a final report within 1 year of study termination.

### Premature study termination

The sponsor or investigator may terminate the study prematurely for a variety of reasons, including unforeseen ethical concerns, insufficient participant recruitment, doubts about the safety of the participants, or alterations in accepted clinical practice that make the continuation of a clinical trial unwise. For patients already enrolled in the study, treatment will be stopped immediately, while patient data will be recorded and observed until the patient leaves the ICU.

If the study has to be terminated prematurely, the regulatory authorities will be informed within 15 days.

### Data management

#### Monitoring

Data will be obtained, recorded, and safely stored in real time by the clinic’s patient monitoring system. Quality assurance will be provided by professional full-time study nurses. Informed consent sheets, randomisation, and case report form completeness will be checked. Study documentation and data will be accessible to auditors/inspectors from the KEK and other regulatory authorities at any time. All parties involved will keep individual data strictly confidential.

#### Data storage

Data will be stored for 10 years in accordance with Swiss law.

### Publication

The authors plan to publish the results of the study in a peer-reviewed journal. Authorship will be granted based on International Committee of Medical Journal Editors (ICMJE) definitions.

## Discussion

Patients undergoing cardiac surgery typically receive a significant amount of intravenous fluids to support circulatory function and counteract the side effects of rewarming after cardiopulmonary bypass [10]. In cardiac surgery patients, the influence of crystalloid solutes on the acid-base status, intracellular and extracellular water content, and plasma electrolyte composition has a substantial impact on organ function and outcome [33–35]. Additionally, changes in vascular permeability and oncotic pressure contribute to fluid leakage [36]. Therefore, these patients typically suffer from various degrees of fluid overload after prolonged ICU stay [10]. Fluid overload has previously been associated with increased mortality and morbidity in various subgroups of critically ill patients [37–39].

Preliminary data from a study conducted recently in our institution showed that fluid balance on the first postoperative day is around plus 6500 ml [40]. Therefore, the exploration of alternative fluid resuscitation strategies for patients after cardiac surgery in order to minimise fluid overload seems of paramount importance.

Previous data suggest that HS might be a safe alternative for fluid resuscitation in patients for whom fluid overload should be avoided [27]. However, data on the use of HS for this indication are scarce: Only five studies have assessed volume status as a primary target criterion after infusion of HS [22, 27, 41–43], with only one study being performed in patients after cardiac surgery [27]. The latter was a randomised controlled double-blind trial involving 72 post-cardiac surgical patients [27]. Endpoints of this study were postoperative weight gain as well as total volume balance when fluid resuscitation with 0.9% saline was compared to a bolus of 4 ml/kg 7.5% saline plus 0.9% saline [27]. The study showed a promising 10% reduction in total volume need, a 50% increase in 1-h urine production, and a 50% reduction of perioperative weight gain [27]. Nonetheless, the results of this study have not been confirmed so far.

In addition, other authors have assessed HS in various settings. Two of these studies investigated HS for preloading before spinal anaesthesia [22, 41]. Veroli and colleagues conducted a study in 30 patients, classified as American Society of Anesthesiologists (ASA) category I, who were undergoing minor orthopaedic surgery under spinal anaesthesia. Patients received HS (5%), NS, or dextrose 5% for fluid preloading (2 mmol sodium/kg) [41]. Preloading with HS resulted in less total intraoperative fluid volume administered [41]. Jarvela and co-workers conducted a randomised double-blind study to evaluate the effects of 7.5% HS on extracellular water volume and hematocrit in patients undergoing arthroscopy or other lower limb surgery under spinal anaesthesia [22]. A total of 40 patients were included in the study; they received 1.6 ml/kg of HS or 13 ml/kg of NS for preloading before spinal anaesthesia [22]. Total infusion volume was markedly lower in the HS group, but haemodynamic indices remained comparable between groups [22]. The authors concluded that HS may be a safe alternative in patients in whom fluid overload should be avoided [22].

Two additional studies targeted patients with volume overload [42, 43]. Paterna and Parrinello performed two randomised controlled trials involving patients with chronic heart failure with New York Heart Association (NYHA) classification III/IV [42, 43]. Patients in respective studies received either HS twice daily with 250 mg furosemide or furosemide only for treatment of fluid overload [42]. Patients in the HS group showed a significant increase in diuresis, reduction in whole body water content, and significant improvement in NYHA class and ejection fraction as assessed by echocardiography [42, 43]. Serum creatinine and blood urea nitrogen (BUN) increased in the control group, whereas they decreased in the HS group [42, 43]. Additionally, hospitalisation time, readmission rate, and mortality rates were reduced in the HS group [42, 43].

We thus are conducting this randomised double-blind controlled clinical trial to test the hypothesis that use of HS results in less total volume infused and less postoperative weight gain when compared to standard of care in post cardiac surgery patients.

### Strengths and limitations

To our knowledge, this is the first randomised controlled trial comparing two different saline solutions (7.3% versus 0.9%) in cardiac surgical patients in the ICU. The preliminary character of the study will allow further hypothesis generation in other areas, such as emergency cardiac surgery or sepsis.

Because this study will be performed at a single tertiary care centre, the external validity is limited. In addition, note that this is a preliminary study, so the number of patients included is limited. A further limitation of our study arises from the exclusion of sick patients.

### Trial status

The trial protocol and schedule are as follows:

- Protocol version: V1.3, March 1, 2018
- 2016–2017: Finalisation of research strategy, finalisation of protocol, approvals
- 2017–2018: Funding obtained
- 2018–2019: First patient enrolled (February 27, 2018); end of trial predicted for the end of 2019
- 2020: Analysis of trial results and publication

## Additional file

Additional file 1: SPIRIT 2013 checklist: recommended items to address in a clinical trial protocol and related documents. (DOC 122 kb)

## Data Availability

The datasets generated and/or analysed during the current study will not be made publicly available in order to protect the participating individuals, but are available from the corresponding author in the case of a reasonable non-commercial request.

## Abbreviations

BMI: Body mass index
ECMO: Extracorporeal membrane oxygenation
eGFR: Estimated glomerular filtration rate
GCP: Good Clinical Practice
HS: Hypertonic saline
IABP: Intra-aortic balloon pump
ICF: Informed consent form
ICU: Intensive care unit
IMP: Investigational medicinal product
KEK: Kantonale Ethikkommission
LVAD: Left ventricular assist device
LVEF: Left ventricular ejection fraction
MAP: Mean arterial pressure
NS: Normal saline
PAC: Pulmonary artery catheter
SOP: Standard operating procedure
